# Recapitulating Actin Module Organization in the *Drosophila* Oocyte Reveals New Roles for Bristle-Actin-Modulating Proteins

**DOI:** 10.3390/ijms22084006

**Published:** 2021-04-13

**Authors:** Ramesh Kumar Krishnan, Raju Baskar, Bakhrat Anna, Natalie Elia, Mandy Boermel, Andreas R. Bausch, Uri Abdu

**Affiliations:** 1 Department of Life Sciences, Ben-Gurion University of the Negev, Beer Sheva 84105, Israel; krishnan@post.bgu.ac.il (R.K.K.); raju@post.bgu.ac.il (R.B.); bakhrat@bgu.ac.il (B.A.); elianat@post.bgu.ac.il (N.E.); 2 National Institute for Biotechnology in the Negev (NIBN), Ben-Gurion University of the Negev, Beer Sheva 84105, Israel; 3 Electron Microscopy Core Facility, European Molecular Biology Laboratory (EMBL), Meyerhofstrasse 1, 69117 Heidelberg, Germany; mandy.boermel@embl.de; 4 Lehrstuhl für Zellbiophysik E27, Technische Universität München, James-Franck-Str. 1, 85748 Garching, Germany; abausch@mytum.de; 5 Center for Protein Assemblies (CPA), Ernst-Otto-Fischer Str. 8, 85747 Garching, Germany

**Keywords:** actin bundles, bristle, *Drosophila*, Fascin, oocyte

## Abstract

The generation of F-actin bundles is controlled by the action of actin-binding proteins. In *Drosophila* bristle development, two major actin-bundling proteins—Forked and Fascin—were identified, but still the molecular mechanism by which these actin-bundling proteins and other proteins generate bristle actin bundles is unknown. In this study, we developed a technique that allows recapitulation of bristle actin module organization using the *Drosophila* ovary by a combination of confocal microscopy, super-resolution structured illumination microscopy, and correlative light and electron microscope analysis. Since Forked generated a distinct ectopic network of actin bundles in the oocyte, the additive effect of two other actin-associated proteins, namely, Fascin and Javelin (Jv), was studied. We found that co-expression of Fascin and Forked demonstrated that the number of actin filaments within the actin bundles dramatically increased, and in their geometric organization, they resembled bristle-like actin bundles. On the other hand, co-expression of Jv with Forked increased the length and density of the actin bundles. When all three proteins co-expressed, the actin bundles were longer and denser, and contained a high number of actin filaments in the bundle. Thus, our results demonstrate that the *Drosophila* oocyte could serve as a test tube for actin bundle analysis.

## 1. Introduction

Parallel actin bundles are composed of tightly packed filaments, all with the same polarity, crosslinked by an actin-bundling protein [1,2,3]. These actin bundles are key components of eukaryotic cytoskeleton structures, such as the brush border of intestinal epithelial cells [4,5,6], stereocilia of hair cells of the vertebrate and ear [7,8], Sertoli cell-spermatid ectoplasmic specialization [9,10], the nurse-cell strut in *Drosophila* eggs [11,12], and insect epidermal cell types, such as bristles and scales [13,14,15]. These actin bundles appear to function in part as scaffolds that help support or stabilize cellular protrusions. In each cytoskeleton structure, the generation of F-actin bundles is tightly controlled by the sequential action of multiple actin-binding proteins [16].

*Drosophila* bristle cells have been used to study actin bundle formation since alterations in their morphology are simple to follow using live imaging. The bristle cytoplasm contains actin filament bundles, which are essential for bristle growth [17,18,19,20]. These actin bundles within the bristle cells are organized in a unique manner, where they are evenly spaced around the outer perimeter of the bristle just inside the membrane, extending from the base of the bristle to its tip. Bristle actin bundles are among the largest such structures in nature, containing more than 500 actin filaments packed together. It has been shown that these bundles in bristles are composed of a series of short modules attached end to end. Accordingly, it has been shown that two actin crosslinker proteins—Forked (human epsin protein homologue) and Singed (the *Drosophila* Fascin homologue, hereafter referred to in the text as Fascin)—are involved in bristle actin bundle formation. It was suggested that Forked functions at early stages in this formation, and then Fascin appears later, displacing Forked to form more regular, more tightly packed filament bundles [21]. In vitro model systems have demonstrated that actin bundles formed by Fascin are limited in size due to an intricate balance of twisting and crosslinking binding energies [22]. Only an additional crosslinking protein enables the formation of thick bundles, as found in bristles [22].

Although Forked and Fascin were found to be the major actin-bundling proteins in bristle formation, it was suggested that there is at least one actin filament-to-membrane connector, and possibly even a third bundling protein, which remains to be identified [23]. One possible candidate for a novel actin bundle protein is Javelin (Jv), which is an actin-associated protein that specifically affects bristle actin formation [24]. Moreover, we demonstrated that Jv is a novel actin-bundling protein; however, further experiments are necessary to understand the bundling propensity of this actin-binding protein.

Recent work from our lab revealed that ectopic expression of a truncated form of GFP-Forked generated a distinct asymmetric actin bundle network in the *Drosophila* oocyte [25]. This localization pattern resembled that reported for the polarized MT network and, indeed, we demonstrated that Forked associates with Short stop and Patronin foci, which assemble non-centrosomal MT-organizing centers. In this study, we decided to use this ectopic Forked-dependent actin bundle network as a model system to study actin bundle formation. Given that Forked is the main actin crosslinker in the *Drosophila* bristle, we tried to understand its interaction with other actin-associated genes that have a role in bristle actin bundle formation, namely, Fascin and Jv. Using a combination of confocal microscopy, super-resolution structured illumination microscopy (SR-SIM), and correlative light and electron microscope analysis (CLEM), we revealed that in the oocyte, Forked and Fascin are sufficient for generating bristle-like actin bundles and that Jv may have a role in actin bundle stabilization and elongation.

## 2. Results

### 2.1. The Combination of Jv, Fascin, and Forked Generate Unique Actin Bundles in the Drosophila Oocyte

**Figure 1:**
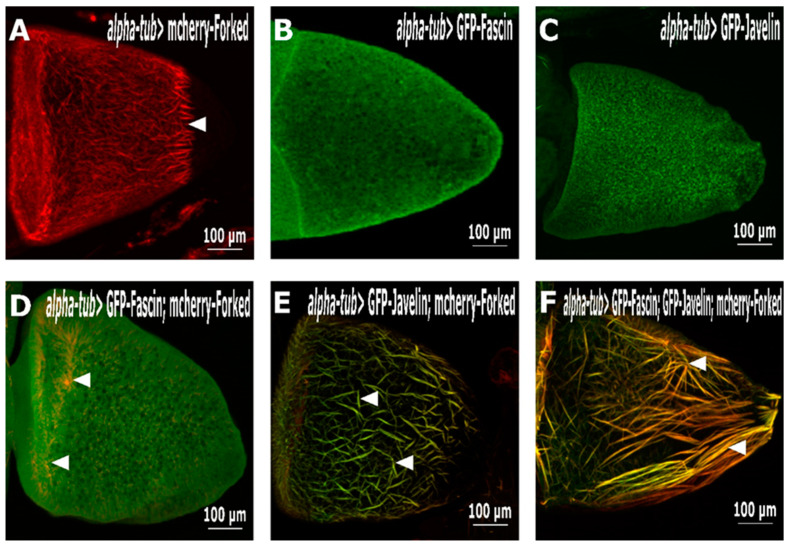
The combination of Jv, Fascin, and Forked generates unique actin bundles in the *Drosophila* oocyte. However, Forked on its own (not Fascin and Javelin) generates asymmetric actin bundles in the *Drosophila* oocyte. Confocal projections of egg chambers from flies ectopically expressing (**A**) mCherry-Forked, (**B**) GFP-Fascin, (**C**) GFP-Javelin, (**D**) GFP-Fascin and mCherry-Forked, (**E**) GFP-Javelin and mCherry-Forked, and (**F**) GFP-Fascin, GFP-Javelin, and mCherry-Forked with alpha-tub. In (**D**), actin bundles are restricted to the anterior side (arrows) of the oocyte, and in (**E**), Jv and Forked decorate the actin bundles (arrows point to one such bundle). In (**F**), combination of the three proteins generates wider and longer actin bundles (arrows point to one such bundle). In all images, the posterior part of the oocyte is on the right.

Previously, we were able to show that Forked generated a distinct ectopic network of an asymmetric network that co-localized with actin (Baskar et al., 2019, Figure 1A). Thus, we decided to use the *Drosophila* ovary as a system to study the combinational effects of bristle-actin-bundling proteins—namely, Forked, Fascin, and Javelin—on actin bundling. In contrast to Forked (Figure 1A, arrowhead shows the asymmetric actin bundles network), expression of Fascin (Figure 1B) or Jv (Figure 1C) revealed that the proteins were distributed throughout the oocyte during mid-oogenesis, with Fascin showing a noticeable accumulation on the oocyte membrane. Next, we generated transgenic fly lines that would allow combinatorial co-expression of Jv, Forked, and Fascin. We showed that none of the single, double, or triple expression combinations affected female fertility (Table 1). We found that the expression of GFP-Fascin with mCherry-Forked affected mCherry-Forked asymmetric network organization. Whereas in the egg chamber from the mid-oogenesis stage, the Forked asymmetric network is restricted to the anterolateral region of the oocyte cortex (Figure 1A), in the egg chamber co-expressing GFP-Fascin with mCherry-Forked, the Forked-Fascin network was restricted to the anterior end of the oocyte (arrowheads in Figure 1D).

Then we tested the effect of expressing GFP-Jv on mCherry-Forked ectopic actin bundle formation, and found that this led to a dramatic change in the actin bundle organization (Figure 1E, Table 2). This time, the actin bundles were much longer and wider (arrowheads in Figure 1E) than in the mCherry-Forked network. The average length (calculated from confocal projections) and width (calculated from the SR-SIM projections) of the ectopic actin bundles in oocytes expressing mCherry-Forked alone (Figure 1A) were 6.17 ± 1.2 µm and 0.25 ± 0.006 µm, respectively (Table 2). In contrast to that, upon additional expressing of Jv, a highly significant increase (*p* < 0.0001) in ectopic actin bundle length (16.1 ± 3.4 µm) and width (0.47 ± 0.01 µm) was evident (Figure 1E, Table 2).

Interestingly, when all three proteins—namely, mCherry-Forked, GFP-Jv, and GFP-Fascin (Figure 1F)—were co-expressed, the bundle networks were much denser than in all other conditions, most notably at the posterior part of the oocyte. In addition, a significant increase (*p* < 0.0001) in ectopic actin bundle length (23.9 ± 1.11 µm) and a significant increase in width (0.67 ± 0.023 µm) was seen (Table 2).

### 2.2. Super-Resolution Microscopy Revealed the Co-Localization of the Actin-Bundling Proteins

**Figure 2:**
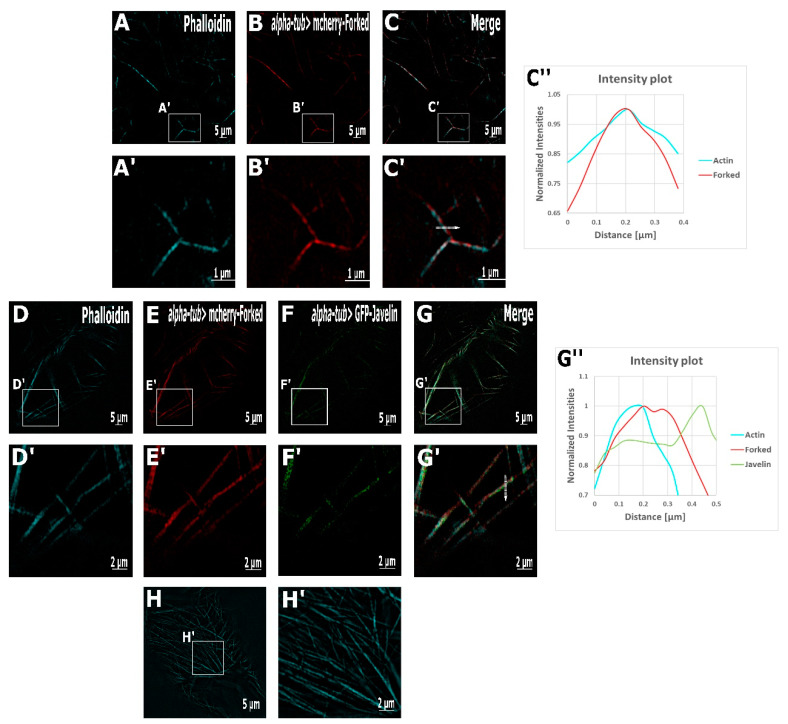
Super-resolution microscopy reveals localization pattern. Super-resolution structure illumination microscopy (SR-SIM) images of actin bundles from flies expressing mCherry-Forked in the oocyte (**A**–**C**). (**A**,**A’**) Phalloidin (actin), (**B**,**B’**) mCherry-Forked, and merged pictures of A and B (**C**,**C’**). Intensity profile of both channels along the actin bundle (**C’’**) normalized using the same color code as SIM-merged images. Actin and Forked are found to be co-localized. SR-SIM analyses of an egg chamber from GFP-Javelin and mCherry-Forked with alpha-tub (**D**–**G**). (**D**,**D’**) Phalloidin, (**E**,**E’**) mCherry-Forked, (**F**,**F’**) GFP-Javelin, and (**G**,**G’**) a merged picture of all three channels. Intensity profile of all the channels along the actin bundle (**G’’**) normalized using the same color code as SIM-merged images quantifies the localization pattern of the proteins. Actin and Forked are found to be co-localized, but Jv is only partially co-localized with Forked and actin. SR-SIM analyses of phalloidin staining from GFP-Fascin-, mCherry-Forked-, and GFP-Javelin-expressing oocytes (**H**,**H’**). As observed in (**H’**), the actin bundles are packed comparatively very tightly close to each other.

To further understand the spatial organization of the actin network, and also to determine the localization of Forked on the ectopic oocyte actin bundles, we conducted a super-resolution structure illumination (SR-SIM) analysis (Figure 2). To quantify the degree of co-localization between actin and Forked, the Pearson’s correlation coefficient was calculated. In oocytes expressing mCherry-Forked alone, actin (Figure 2A) was highly co-localized with Forked (Figure 2B), with a Pearson’s correlation coefficient > 0.9 (*p* value < 0.05, Figure 2C’,C’’). Using SR-SIM, the organization of actin bundles at the anterior end of the oocyte could not be resolved.

In ovaries expressing mCherry-Forked and GFP-Jv, our SR-SIM analysis revealed that the actin bundles were denser. Indeed, the distance between actin bundles in oocytes expressing both mCherry-Forked (Figure 2E) and GFP-Jv (Figure 2F) was significantly (*p* < 0.01) reduced to 0.73 ± 0.06 µm (Figure 2G) compared to the average.

Distance between actin bundles was 2.18 ± 0.15 µm (Table 2) in oocytes expressing mCherry-Forked alone (Figure 2C). Moreover, using SR-SIM, we found that that actin and Forked were still co-localized (Pearson’s correlation coefficient was above 0.7 with a *p* value < 0.05), but interestingly, Jv was found to only partially decorate the actin-Forked filaments, showing a shift towards the sides of the filament (Figure 2G’,G’’). Indeed, the Pearson’s correlation coefficient for Jv with reference to both actin and Forked was above 0.4, suggesting a low-to-moderate correlation; in other words, Jv was found to be partially co-localized either with actin or Forked.

We also found that in oocytes expressing Forked, Fascin, and Jv (Figure 2H,H’), the actin bundles were tightly packed, with the distance between the filaments being just 0.34 ± 0.15 µm.

### 2.3. Analysis of the Internal Organization of the Ectopic Oocyte Actin Bundles Generated by Forked, Javelin, and Fascin by CLEM

**Figure 3:**
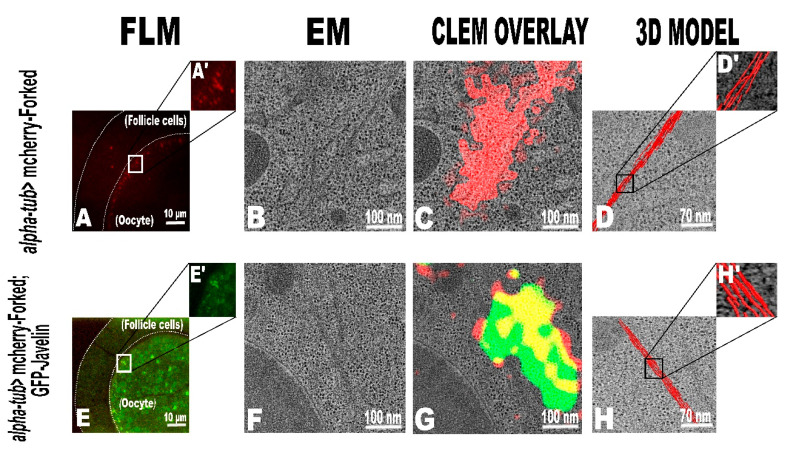
On-section correlative light and electron microscope analysis (CLEM) of *Drosophila melanogaster* oocytes overexpressing mCherry-Forked and mCherry-Forked with GFP-Javelin. Cross-section of an egg chamber for flies ectopically expressing mCherry-Forked (**A**) or mCherry-Forked and GFP-Javelin (**E**) with alpha-tub, visualized by fluorescence microscopy (FLM) of an in-resin-retained fluorescence signal. Fluorescently labeled structures of interest i.e., highlighted in (**A’**,**E’**) were identified in the transmission electron microscope by overlaying prominent landmarks in both FLM and EM imaging modalities and subjecting them to high-resolution dual-axis electron tomography. Per condition, 25 tomograms were acquired. (**B**,**F**) represent a tomographic slice for each condition (the same conditions were also done for Figure 4 and Figure 5). After tomogram reconstruction, a CLEM overlay of both imaging modalities was performed with high accuracy (**C**,**G**). A 3D model representation of actin filaments forming bundles (red) was performed manually in Amira. The overexpression of mCherry-Forked (**D**,**D’**) and mCherry-Forked with GFP-Javelin (**H**,**H’**) forms single thick ectopic actin bundles (from ~4 to 5 actin filaments per bundle).

**Figure 4:**
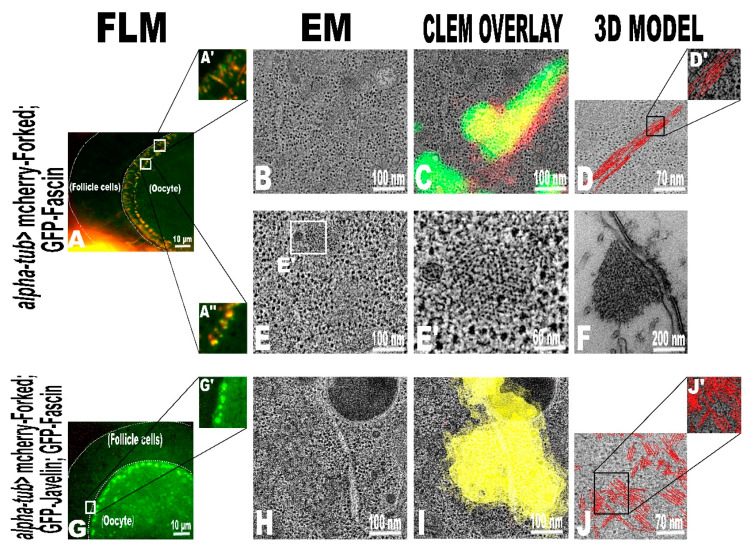
On-section CLEM of *Drosophila melanogaster* oocytes overexpressing mCherry-Forked with GFP-Fascin and mCherry-Forked with GFP-Fascin and GFP-Javelin. Cross-section of an egg chamber for flies ectopically expressing mCherry-Forked with GFP-Fascin (**A**) and mCherry-Forked, GFP-Fascin, and GFP-Javelin (**G**,**G’**) with alpha-tub visualized by fluorescence microscopy (FLM) of an in-resin-retained fluorescence signal. (**B**,**E**,**H**), represent a tomographic slice for each condition. After tomogram reconstruction, a CLEM overlay of both imaging modalities was performed with high accuracy (**C**,**I**). In this condition (**A**), the actin bundles run in both longitudinal (**A’**) and transverse (**A’’**) fashion. The overexpression of mCherry-Forked with GFP-Fascin (**D**,**D’**) forms thick ectopic actin bundles (~9 actin filaments per bundle) as shown in the longitudinal section. On the other hand, (**E**,**E’**) represents a tomographic slice with a cross-sectional actin bundle of about ~95 actin filaments in one cross-sectional area on the bundle. Interestingly, the cross-section of the actin bundle resembles the actin bundles occurring in the cross-section of a *Drosophila* bristle (**F**) during elongation. The overexpression of mCherry-Forked, GFP-Javelin, and GFP-Fascin (**J**,**J’**) forms a dense actin filament network. Many parallel actin bundles (~8 actin filaments in each bundle) are organized in large patches. (**J**,**J’**) show the segmentation of actin filaments in just one tomogram slice (because of bundle overcrowding when all the slices were included).

**Figure 5:**
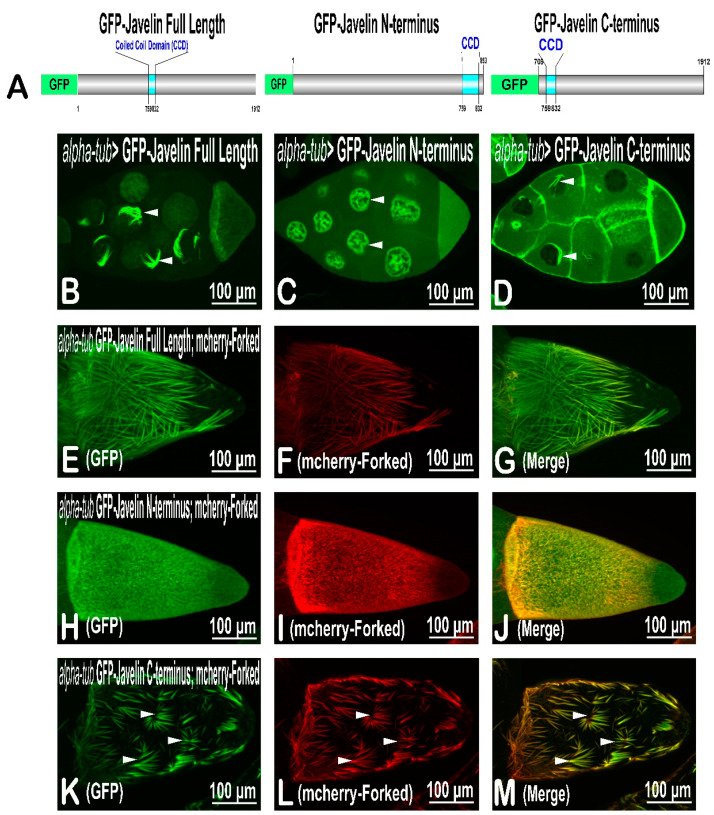
Structure–function analysis of Javelin protein. (**A**) Schematic representation of the domain structures of Javelin that were used to generate transgenic flies: Javelin full length, Javelin N-terminus, and Javelin C-terminus. Each contains the coiled–coil domain (CCD) and is fused to EGFP at its N-terminus end. (**B**–**D**) Confocal projections showing the localization pattern of Jv-full length (**B**), Jv N-terminus (**C**), and Jv-C-terminus (**D**) in *Drosophila* oocytes upon expression with alpha-tub. In all images, the posterior part of the oocyte is on the right. Full-length Jv generates thick brush-shaped actin bundles around the nurse-cell nucleus (pointed arrows), whereas the Jv-N-terminus part is found inside the nurse-cell nuclei. Jv C-terminus generates actin bundles around the nurse-cell nucleus. Confocal projections of egg chambers from flies ectopically expressing GFP-Javelin and mCherry-Forked (**E**–**G**), GFP-N-terminus Javelin and mCherry-Forked (**H**–**J**), and GFP-C-terminus Javelin and mCherry-Forked (**K**–**M**) with alpha-tub. Expression of GFP-N-terminus Javelin (**H**) suppresses mCherry-Forked actin bundle activity (**I**). Expression of GFP-C-terminus Javelin (**K**) affects mCherry-Forked actin bundle activity (**L**); instead of creating a directly aligned network, as seen with full-length Javelin (**E**–**G**), the actin bundles form radiating masses.

To better understand and characterize the internal ultrastructure of actin bundles in 3D, we used correlative light and electron microscope analysis (CLEM). *Drosophila* ovaries expressing mCherry-Forked alone, GFP-Jv and mCherry-Forked, GFP-Fascin and mCherry-Forked, GFP-Jv, and GFP-Fascin were subjected to high-pressure freezing and thin sectioning. The 300 nm sections were stained by toluidine blue (Supplemental Figure S1) to ensure that the sections comprised oocytes and not nurse cells. They were then first visualized by fluorescence microscopy to locate areas of interest for electron tomography analysis, followed by 3D reconstruction of the actin bundles (Figure 3, Figure 4 and Figure 5).

In ovaries expressing mCherry-Forked, the bundles were composed of an average of 4 ± 0.16 filaments, and the average space between them was 11.7 ± 0.5 nm (Figure 3D, Table 3, Supp. Movie S1). Thus, the total width of the actin bundles was 47.8 ± 2.6 nm. In ovaries expressing mCherry-Forked and GFP-Jv, similar actin bundles were identified (Figure 3H), with an average number of 5 ± 0.22 filaments in each bundle. The space between the filaments was 10.3 ± 0.6 nm, with a total width of 52.6 ± 2.3 nm (Table 3, Supp. Movie S1). Thus, although our SR-SIM analysis showed that there was an increase in the width of the actin bundles upon overexpression of mCherry-Forked and GFP-Jv, our CLEM analysis revealed no change in the width and number of the actin filaments within the bundle compared to oocytes expressing mCherry-Forked.

In sections from ovaries expressing both mCherry-Forked with GFP-Fascin, actin bundles at the anterior end of the oocyte could be detected both longitudinally (Figure 4B) and in cross-section (Figure 4E, Supp. Movie S1). Measuring the number of actin filaments of each of the longitudinal sections revealed a dramatic and highly significant increase in the number of actin filaments (Figure 4D, 9 ± 0.37) compared to mCherry-Forked alone (Figure 3D, 4 ± 0.16, Supp. Movie S1). The distance between the actin filaments was 10.4 ± 0.3 nm, and their total width equaled 81.1 ± 4.2 nm. Interestingly, measuring the average number of individual actin filaments at each single cross-sectional area showed that it contained up to 95 ± 9.23 (Figure 4E’). Moreover, closer examination revealed that these actin bundles geometrically resembled those corresponding to the ones seen in the cross-section of a *Drosophila* bristle during elongation (Figure 4F). We could not correctly measure the distance between the actin filaments by conventional room temperature EM preparations; thus, further analysis using cryo-EM is needed.

In addition, a dramatic change in actin bundle spatial organization was noticeable in ovaries expressing all three proteins (Figure 4J; Supp. Movie S1). A denser actin bundle network was primarily found as small, crowded patches in many areas of the cryo-sections. The orientation of these patchy bundles was arbitrary, and they were seen to extend in all directions. We found that the average number of actin filaments in each actin bundle (8 ± 0.1) with space between them was 10.9 ± 0.3 nm, and their total width was 75.1 ± 3.7 nm. 

To conclude, our results demonstrate that the *Drosophila* oocyte could serve as a model tissue to recapitulate bristle-like actin module organization. Moreover, this model system allowed us to reveal a new function for Fascin and Forked, confirming the necessity of combining two crosslinking proteins to obtain thicker bundles, as predicted from an in vitro model system [22].

### 2.4. Structure–Function Analysis of Javelin Protein

At this stage, we decided that we could also use this tool for further structure–function analysis of the Jv protein. Structure analysis showed that Jv consists of 1912 amino-acid residues with only one functional domain—the coiled–coil domain (CCD)—located between amino acids 759 and 832 (Figure 5). A structure–function analysis was performed to understand more about the Jv function and its role in actin bundling. We showed that the GFP-fused Jv protein, ectopically expressed in the germline, is found at the anterior region of the oocyte and in the nurse cells. In addition, the protein forms an ectopic actin network close to the nurse-cell nuclei and is found at their ring canals (Shapira et al., 2011; Figure 5B). We cloned both the N-terminal part (1–832 aa) and the C-terminal part (759–1912 aa) fused to the EGFP protein; thus, each of the truncated forms included the CCD domain. We found that when the Jv N-terminus was expressed in the germline, the GFP-fused truncated protein was within the nurse-cell nucleus (Figure 5C). On the other hand, the C-terminus truncated Jv protein was found to form an ectopic actin network close to the nucleus, similar to that of the full-length Jv, and it was also found at the nurse-cell ring canals (Figure 5D).

Next, we examined the ability of each of the GFP-Jv fused truncated proteins to generate actin-bundle networks with Forked protein. We found that, in contrast to the expression of Forked protein with full-length Jv, where long actin bundles could be detected in the oocyte (Figure 5E–G), co-expression of the Jv N-terminus with Forked protein suppressed Forked actin bundle activity (Figure 5H–J). On the other hand, Jv C-terminus truncated protein co-expression with Forked produced unique patterned actin bundles (Figure 5K–M). Most noticeably, while actin bundles in co-expression of Forked and full-length Jv protein oocytes were directly aligned, the actin bundles in oocytes from flies expressing Jv C-terminus truncated protein with mCherry-Forked formed radiating masses.

## 3. Discussion

### 3.1. The Drosophila Ovary as a Model Tissue to Study Bristle Actin Bundle Formation

Using in vitro studies helps us to understand the mechanism of actin bundle formation [2,26]. These studies illustrate the physical, chemical, and biomechanical driving forces that generate these unique structures. In vitro studies require purified actin proteins, as well as purified proteins of interest. In our attempts to understand the function of Jv and Forked proteins in constructing actin bundles, we encountered one of the most common problems in in vitro studies: the inability to express and purify these proteins from either bacteria, insect, or mammalian cells. Previously, such studies on Fascin [27,28,29,30], Forked [30,31], and Jv mutant flies [24] revealed only part of these gene functions. To better understand the function of these proteins, we decided to use the *Drosophila* ovaries as a model. Our previous study demonstrated that the expression of Forked protein in *Drosophila* ovaries generates a unique actin-bundle network [25]. In this study, we demonstrate that the *Drosophila* ovary could serve as a model tissue to study bristle actin bundle formation for the following reasons: (1) Generating actin bundle networks that can be clearly analyzed by several methods is specific to the oocyte, and our study revealed dramatic and informative changes in actin bundle networks upon combined expression of proteins. (2) Ectopic expression of any single protein or combination of proteins did not affect the organism’s fertility or oocyte development. In addition, the structure of the oocyte remained intact despite the heavy protein load. (3) Our attempts to repeat these results in other cell types, such as follicle cells, Schneider cells (*Drosophila* embryonic cells), and salivary glands failed to generate similar results. (4) Using confocal microscopy, SR-SIM, and CLEM allow us to determine the organization of the actin bundles in the oocyte and also to analyze the bundles on the ultrastructure level. (5) Future studies using genetic tools such as loss and gain of functions could easily be used in the ovaries. Finally, (6) the findings in this study shed new light on the function of two proteins, Fascin and Jv, in actin bundle formation. To conclude, we have demonstrated that the *Drosophila* oocyte could serve as a model tissue to recapitulate bristle-like actin module organization, which opens a new window on understanding the mechanism of other bristle-actin-associated proteins.

### 3.2. Fascin Acts Not Only in Hexagonal Packing of Actin Bundles, But Also Enhances Forked Actin Crosslinking Activity 

Genetic and biochemical studies on the role of Fascin and Forked in bristle development have generated a model to describe the way these two actin crosslinkers act in bristle actin bundle formation [18,21]. It was suggested that Forked and Fascin act in a sequential action [32]. In the first stages of bristle development, Forked is responsible for the crosslinking of tiny actin filament bundles at the newly emerging bristle tip. Forked protein also acts to aggregate these tiny bundles into larger membrane-associated bundles. In the next step, Fascin, by Forked facilitation, enters the bundles to perform the final crosslinking to generate tight hexagonally packed actin bundles. The functions of these two proteins are spatially, temporally, and molecularly regulated. Generation of bristle actin bundles is controlled spatially from the tip region [33], by the level of Forked protein [30], and by the activation of Fascin protein [18]. This tight regulation could not be achieved in our ovary system. Still, our results support one aspect of the suggested model, showing that the addition of Fascin protein into actin bundles formed by Forked generates tightly bundled actin filaments that resemble the geometrical structure (close hexagonal packaging) of bristle actin bundles. Moreover, the dramatic increase in actin filament number upon the addition of Fascin into Forked-made actin bundles reveals a new and not yet described function for Fascin in generating actin bundles, namely enhancing Forked actin crosslinking activity. This underlines the physical basis of the synergistic role of the two crosslinking proteins: where the necessary twist of filaments for hexagonal packing limits the bundle growth, it can be overcome by the addition of a second crosslinking protein [22].

### 3.3. Jv May Have a Role in Actin Bundle Compactization and Elongation

Jv is a spontaneous mutation that was identified in 1947. Previous work from our lab demonstrated that Jv is a novel gene that encodes an actin-associated protein [24]. The only phenotype associated with the Jv mutant is defects in bristles, where they do not taper like bristles in WT flies—instead, presenting a small enlargement before the tip. Our studies revealed that in Jv mutants, bristle actin bundles are affected where the loss of actin filaments within the bundles affects their triangular structure [24]. Still, the molecular function of Jv in bristle actin bundle formation was unclear. In this study, we found that the additional expression of Jv protein in the oocyte to either ectopic expression of Forked, or Forked and Fascin, resulted in longer actin bundles and elevated the density of the actin bundles within the oocyte. On the other hand, whereas previously our confocal microscopy analysis suggested that Jv is co-localized with actin, our SR-SIM analysis revealed that it is only partially co-localized with actin and Forked. What could be the function of Jv in actin bundle formation? We found that ectopic expression of Jv with either Forked, or Forked and Fascin, led to a dramatic increase in the compactization of actin bundles in the oocyte as the distance between the actin bundles decreased. There are several options for explaining this observation. The first is that Jv stabilizes Forked-generated actin bundles; thus, we could detect more of them close to each other. The other option is that Jv, either directly or indirectly, leads to actin bundle aggregation. That the actin bundles, in Jv mutant bristles, lack a proper triangular structure due to the loss of actin filaments within the bundles supports the possibility that Jv is required for actin bundle stabilization. Our study also reveals another possible function of Jv in actin bundle construction. We found that it significantly enhances the length of the actin bundles. This may suggest that Jv is required for actin bundle elongation. In support of this possibility, our previous results showed that the actin bundles in the Jv mutant bristle were shorter and disordered, and ran in different directions along the bristle shaft, rather than straight, as found in the WT bristle. That Fascin can regulate actin elongation factors, specifically Enabled (Ena) processivity [34], suggests that Jv may also stabilize Fascin in bundles, thus increasing Ena processivity and leading to filament elongation.

The presented results may lead to a better understanding of the molecular organization of higher-order structure formation processes.

## 4. Materials and Methods

### 4.1. Drosophila Stocks

The following transgenic flies were used: GFP-Javelin [24], mCherry-Forked [25], GFP-Fascin, and mCherry-Fascin [35]. Germline expression was performed with P{*matα4*-GAL4-VP16} V37 (herein referred to as alpha-tub) obtained from the Bloomington Stock Center.

### 4.2. Fertility Assay

Three virgin females of the respective genotypes were mated with two wild-type (WT) males in a vial containing yeast for two days. Matings were performed in triplicate for each genotype. The flies were transferred to new vials containing fresh yeast for one day to lay eggs. The flies were discarded, and the progeny resulting from the eggs after ten days at 25 °C were collected and counted. The progeny per female and the average number and standard deviation of progeny per genotype were calculated from each vial. Finally, a percentage of relative fertility was calculated [36].

### 4.3. Confocal Microscopy

*Drosophila* ovaries were dissected in PBS, fixed for 10 min in 4% PFA, and washed 3× times in PBS with 0.1% Tween. The ovarioles were transferred onto a glass slide with citifluor antifadent mountant solution (Electron Microscopy Sciences) and gently separated for better imaging. All images were taken with an Olympus FV1000 laser-scanning confocal microscope. The length and width of the actin bundles were measured using Fiji/ImageJ software with the straight-line tool. This tool allows the creation of line selections, followed by calculation of the length of the same.

### 4.4. SR-SIM Imaging

*Drosophila* ovaries were dissected in PBS and fixed for 10 min in 4% PFA. All the samples were stained with Alexa Fluor 633 phalloidin dye (Molecular Probes, Oregon, USA) to stain the endogenous actin and washed 3× times in PBS with 0.1% Tween. The ovarioles were transferred onto a glass slide with citifluor antifadent mountant solution and gently separated for better imaging. Thin z-stacks of high-resolution images were collected using an ELYRA PS.1 microscope (Carl Zeiss MicroImaging, Oberkochen, Germany) at three rotations in five phases. Images were then reconstructed using ZEN software (Carl Zeiss MicroImaging, Oberkochen, Germany) based on the structured illumination algorithm developed by Heintzmann and Cremer [37]. All measurements were performed on reconstructed super-resolution images of single z-sections using ZEN software. 

All images were subjected to channel alignment using four-color beads, and the measurements were performed on reconstructed super-resolution images of single z-sections with ZEN software. The distance between the actin bundles was measured using Fiji/ImageJ software with the straight-line tool. 

Co-localization of fluorescence signals was analyzed from their intensity profiles with the ZEN software. The co-localization value (Pearson’s coefficient) was manually calculated from five representative images for each genotype in Microsoft Excel.

### 4.5. On-Section CLEM

#### 4.5.1. Sample Preparation

*Drosophila melanogaster* ovarioles were dissected from ovaries in Schneider’s medium (+10% FCS and 1% insulin). The ovarioles were transferred to the medium containing an additional 20% Ficoll (PM 70), and Stage 10 oocytes were separated for cryo-immobilization. High-pressure freezing was performed with HPM-010 (Abra-Fluid), where two oocytes were cryo-immobilized in a 200-μm deep type-A carrier (Wohlwend) imbibed with 1-hexadecene and covered with 20% Ficoll (PM 70) in Schneider’s medium as a cryo-protectant. All samples were further processed by freeze-substitution (FS) and flat-embedding in a temperature-controlling device (EM-AFS2, Leica Microsystems). FS was carried out at −90 °C for 52 h with 0.1% (*w*/*v*) uranyl acetate in glass-distilled acetone (EMS). The temperature was then raised to −45 °C (3.5 °C/h), and samples were further incubated for 5 h. After rinsing in acetone, samples were infiltrated with increasing concentrations (10, 25, 50, and 75%; 4 h each) of Lowicryl HM20 resin (EMS) in acetone, while the temperature was further raised to −25 °C. 100% Lowicryl was exchanged three times in 10 h steps, and samples were UV polymerized at −25 °C for 48 h, after which the temperature was raised to 20 °C (5 °C/h), and UV polymerization continued for 6 h. Cross-section 300 nm thick of the anterior to the central part of the oocyte were cut with an ultra-microtome (UC7, Leica) and a diamond knife (ultra semi, DiATOME) and picked up on carbon-coated 200-mesh copper grids (S160, Plano). Targeting of the oocyte was carried out using toluidine blue sections. The Stage 10 oocyte was readily distinguishable from the nurse cells by certain anatomical landmarks such as peripheral follicle cells and huge yolk particles.

#### 4.5.2. Fluorescence Microscopy Imaging

Ultramicrotomy and acquisition of the in-resin-retained fluorescence within the sections are best performed on the same day to avoid bleaching of the fluorescence. Thus, the fluorescence microscopy (FM) imaging of the sections was carried out as previously described [38] using a wide field fluorescence microscope (Olympus IX81) equipped with an Olympus PlanApo 100X 1.40 NA oil immersion objective.

#### 4.5.3. Electron Tomography

Dual-axis tilt series (1° increment, −60° to 60°) of the areas of interest were acquired using an FEI TECNAI F30 TEM operated at 300 kV and a fast Gatan OneView 4K camera. Tomograms were reconstructed at a final voxel size of 1.55 nm using patch tracking and weighted-back projection algorithms of the software package IMOD [39]. 

#### 4.5.4. Correlation and Segmentation

Correlation between light and electron micrographs was carried out with the plugin ec-CLEM [40] of the software platform Icy [41]. The coordinates of pairs of corresponding features in the two imaging modalities (autofluorescent uranyl-acetate-stained yolk granules, mitochondria, vesicles) were used to calculate a linear transformation that allowed mapping the coordinates of the fluorescent spots of interest to overlay them on the electron micrograph. Electron tomograms were displayed and analyzed using the IMOD software package [39]. The interfilament distance and bundle width were also measured using IMOD. Manual segmentation of the actin filaments was performed within Amira visualization software [42].

### 4.6. Constructs and Transgenic Flies

The GFP gene was cloned into plasmid pUASp-attB using the *Kpn*I and *Xba*I restriction sites by Gibson assembly (hereafter, the constructs are designated as plasmids pUASp-GFP). DNA encoding Jv N-terminus (residues 1–853) and Jv C-terminus (residues 706–1912) were then cloned into plasmid pUASp-GFP using the *Xba*I restriction sites. The following primers were used: Jv N-terminus Forward primer 5′-ATGGGCAACGGATATTTTCG-3′, Jv N-terminus Reverse primer 5′-AGTCGTCCTCAGCCTCTTCC-3′, Jv C-terminus Forward primer 5′-TGGTCACTGCCAACAAGTC-3′, and Jv C-terminus Reverse primer 5′-TACATTTTGTCATCCAGGCTG-3′. P-element-mediated germline transformation of these constructs into the attP site (ensuring equal expression levels of each construct) was carried out by BestGene.

### 4.7. Data Analyses

Actin bundle lengths were measured by confocal microscopy, and their width and the distance between them were measured by SR-SIM. The number of actin filaments within the bundles, the inter-actin-filament distance within the bundles, and their total width were measured by CLEM. Quantitative data are expressed as mean ± standard error of the mean (SEM). All the statistical analyses were performed using a one-way ANOVA, and *p* values ≤ 0.05 were considered significant for all analyses. The statistical significance was checked with a pairwise post hoc Tukey HSD. All the statistical analyses were performed using STATISTICA, version 10.

## Figures and Tables

**Table 1:** Overexpression of Forked, Fascin, and Jv and their combinations in the germline using *alpha-tub*-GAL4 do not affect female fertility. Tukey’s test for post hoc analysis shows that the percentage of relative fertility had no significant difference when compared statistically to the control group. ^a^ Same letter in the column indicates no significant statistical difference among the groups.

S. No.	Genotype	Average No. of Progeny from 24 h Egg Collection	% Relative Fertility
1	Kr/CyO; *alpha-tub*	87 ± 6.1	100 ^a^
2	mCherry-Forked/CyO; *alpha-tub*	77 ± 1.7	88.5 ^a^
3	GFP-Fascin/CyO; *alpha-tub*	74.6 ± 7.6	85.8 ^a^
4	GFP-Javelin/CyO; *alpha-tub*	76 ± 5.1	87.3 ^a^
5	GFP-Fascin/GFP-Javelin; *alpha-tub*	73.3 ± 9.3	84.2 ^a^
6	GFP-Fascin/CyO; mCherry-Forked/*alpha-tub*	74 ± 4.7	85.1 ^a^
7	GFP-Javelin/CyO; mCherry-Forked/*alpha-tub*	72.3 ± 9.2	83.1 ^a^
8	GFP-Fascin/GFP-Javelin; mCherry-Forked/*alpha-tub*	70.6 ± 8.1	81.2 ^a^

**Table 2:** Table showing the actin bundle lengths from confocal projections. Tukey’s test for post hoc analysis shows that a, b, and c are statistically significant to each other (*p* < 0.01). The table also shows the width and inter-actin bundle distance from SR-SIM projections. Tukey’s test for post hoc analysis for both inter-bundle distance and width shows that a, b, and c are statistically significant to each other (*p* < 0.01). Seven oocytes were taken for the analysis, and ten actin bundles from each were used for measurement. ^a–c^ Different letters in all the columns indicate significant statistical difference among the groups.

		Confocal Projections	SR-SIM Projections	
S. No.	Genotype	Length (µm)	Interbundle Distance (µm)	Width (µm)
1	mCherry Forked	6.17 ± 1.2 ^a^	2.18 ± 0.15 ^a^	0.25 ± 0.006 ^a^
2	mCherry Forked_GFP Javelin	16.17 ± 3.4 ^b^	0.73 ± 0.06 ^b^	0.47 ± 0.017 ^b^
3	mCherry Forked_GFP Fascin_ GFP Javelin	23.96 ± 1.11 ^c^	0.34 ± 0.15 ^c^	0.67 ± 0.023 ^c^

**Table 3:** Table showing the width and inter-actin-filament distance measurements from the tomograms of ovaries expressing mCherry-Forked, mCherry-Forked with GFP-Jv, mCherry-Forked with GFP-Fascin, and mCherry-Forked with GFP-Jv and GFP-Fascin under CLEM conditions. Tukey’s test for post-hoc analysis shows that a and b are statistically significant to each other (*p* < 0.01), whereas groups 2, 3, and 4 show no significant difference in terms of interfilament distance. Eight oocyte sections were taken for the analysis, and 25 tomograms were acquired. Four actin bundles from each were used for measurement. ^a,b^ Different letters in the column indicate significant statistical difference among the groups.

S. No.	Genotype	Interfilament Distance (nm)	Total Width (nm)
1	mChFk	11.7 ± 0.5 ^a^	47.8 ± 2.6
2	mChFk_GFPJv	10.3 ± 0.6 ^b^	52.6 ± 2.3
3	mChFk_GFPFascin	10.4 ± 0.3 ^b^	81.1± 4.2
4	mChFk_GFPJv_GFPFascin	10.9 ± 0.3 ^b^	75.1 ± 3.7

## Data Availability

Not applicable.

## Supplementary Materials

The following are available online at https://www.mdpi.com/article/10.3390/ijms22084006/s1, Figure S1: Toluidine blue-stained sections of an ovariole expressing GFP-Fascin and mCherry-Forked. Movie S1, CLEM animation: Animation showing a 3D-EM rendering of ectopic actin bundles within the *Drosophila* oocyte analyzed by CLEM (Amira software).
