# Supplementary material for: Recapitulating Actin Module Organization in the Drosophila Oocyte Reveals New Roles for Bristle-Actin-Modulating Proteins
Source: Int J Mol Sci. 2021 Apr 13;22(8):4006. doi: 10.3390/ijms22084006 (PMC8070096; doi:10.3390/ijms22084006)
Supplement: Supplementary file 1 [file ijms-22-04006-s001.zip › ijms-1163135-supplementary/ijms-1163135-captions.pdf]

Figure S1: (A–C) Toluidine blue-stained sections of an ovariole expressing GFP-Fascin and mCherry-Forked. (A) shows the ovariole at a distance of −55 µm from the imaged area in Figure 4B, and the nurse cells' nuclei are still visible. (B) shows the ovariole at a distance of −35 µm from the area of interest. The nurse cell nuclei are still visible. (C) No nurse cell nuclei are seen anymore at a distance of −10 µm from the area shown in Figure 4B. Its substantial yolk granules clearly distinguish the oocyte, and the oocyte is surrounded by the follicle cells. Once the oocyte is reached, the sections are taken for subsequent image acquisition. (D) represents one of the many areas obtained by TEM imaging from the oocyte at the anterior site of the oocyte (for all the conditions shown in the results),

Movie S1: CLEM animation: Animation showing a 3D-EM rendering of ectopic actin bundles (red) within the *Drosophila* oocyte analyzed by CLEM (Amira software). Overexpression of mCherry-Forked forms ectopic actin bundles consisting of ~4–6 filaments at the oocyte. While mCherry-Forked was co-expressed with GFP-Javelin, actin bundles containing ~4–6 filaments were generated. Co-expression of mCherry-Forked with GFP-Fascin yielded actin bundles that were oriented in two different directions: longitudinal and transverse. The longitudinal bundles were made up of around 9–10 filaments each, whereas the transverse bundles consisted of ~95–100 actin filaments. Finally, when all three proteins were expressed, a dramatic increase in actin bundle density was observed. Each bundle consisted of around 7–9 actin filaments, and the bundles were packed very close to each other.
