# Supplementary figures and images for: Recapitulating Actin Module Organization in the Drosophila Oocyte Reveals New Roles for Bristle-Actin-Modulating Proteins
Source: Int J Mol Sci. 2021 Apr 13;22(8):4006. doi: 10.3390/ijms22084006 (PMC8070096; doi:10.3390/ijms22084006)

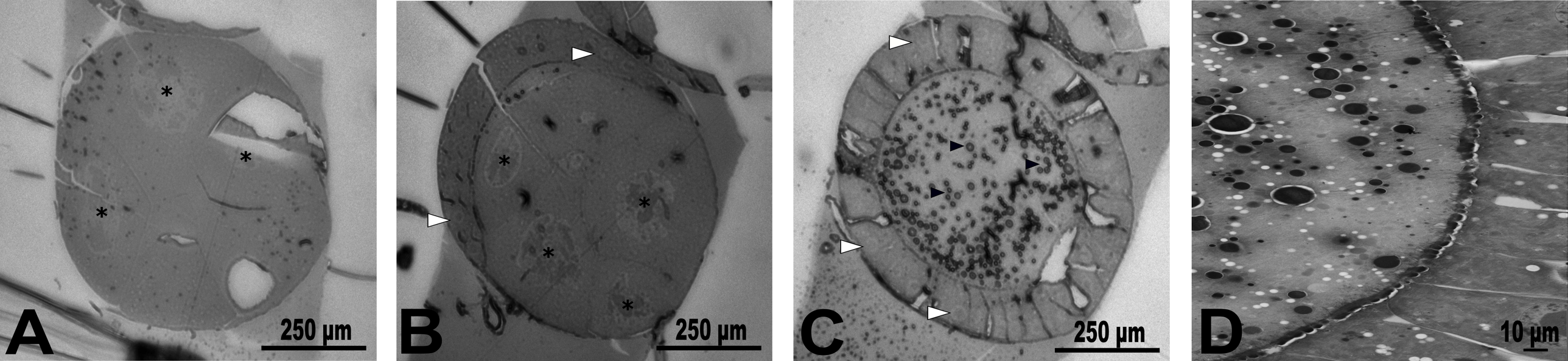

Supplement: Supplementary file 1 [file ijms-22-04006-s001.zip › ijms-1163135-supplementary/ijms-1163135.jpg]
